# Horizontal Transfer of a Plant Transposon

**DOI:** 10.1371/journal.pbio.0040005

**Published:** 2005-12-20

**Authors:** Xianmin Diao, Michael Freeling, Damon Lisch

**Affiliations:** **1**Department of Plant and Microbial Biology, University of California at Berkeley, Berkeley, California, United States of America; Cold Spring Harbor Laboratory, United States of America

## Abstract

The majority of well-documented cases of horizontal transfer between higher eukaryotes involve the movement of transposable elements between animals. Surprisingly, although plant genomes often contain vast numbers of these mobile genetic elements, no evidence of horizontal transfer of a nuclear-encoded transposon between plant species has been detected to date. The most mutagenic known plant transposable element system is the *Mutator* system in maize. *Mu*-like elements (MULEs) are widespread among plants, and previous analysis has suggested that the distribution of various subgroups of MULEs is patchy, consistent with horizontal transfer. We have sequenced portions of MULE transposons from a number of species of the genus *Setaria* and compared them to each other and to publicly available databases. A subset of these elements is remarkably similar to a small family of MULEs in rice. A comparison of noncoding and synonymous sequences revealed that the observed similarity is not due to selection at the amino acid level. Given the amount of time separating *Setaria* and rice, the degree of similarity between these elements excludes the possibility of simple vertical transmission of this class of MULEs. This is the first well-documented example of horizontal transfer of any nuclear-encoded genes between higher plants.

## Abstract 2

Sequencing and analysis of MULE transposons and their surrounding genomic regions from closely related grass species and rice provides evidence of horizontal transfer in plants.

## Introduction

Horizontal transfer can be defined as the process by which genes can move between reproductively isolated species. It is far more frequent than was once thought, particularly among prokaryotes [1], to the extent that it has complicated analysis of bacterial evolution [2]. Like many transfers between bacteria, most well-documented transfers detected to date between higher eukaryotes involve movement of transposable elements [3]. The best-documented examples involve the transfer of *P* [4] and *mariner* elements [5] between animal species. In contrast, no case of horizontal transfer of a nuclear-encoded plant gene has been reported. Recently, systematic analysis of mitochondrial genes and group I mitochondrial introns has revealed that they have been subject to frequent horizontal transfer between plant species [6–8], suggesting that there is no fundamental barrier to gene flow between reproductively isolated plant species. Interestingly, despite the vast numbers of transposable elements found in most plant nuclear genomes and the demonstrated propensity of these elements to move between animal species, no evidence for horizontal transfer of nuclear-encoded plant transposons has been detected. This is in part due to the lack of comprehensive surveys of these elements in a large number of plants. However, extensive phylogenetic analysis of *haT* [9,10] and *mariner* [11] transposons in a variety of plant species has been consistent with vertical, rather than horizontal, transmission of these elements in plants. The same appears to be the case for CACTA elements [12] and the *Ty1-copia* group of retrotransposons [13] as well. The case of *mariner* elements is particularly surprising given that *mariner* elements in metazoans are particularly prone to horizontal transfer, to the extent that such transfer has been hypothesized to be an integral part of the success of this class of elements [14].

*MuDR* is an unusually active and mutagenic maize class II (DNA intermediate) transposon. It is the autonomous member of a family of transposable elements in maize, all of which share the same 200–base pair (bp) terminal inverted repeats, but each of which contains a unique internal sequence. *MuDR* encodes two genes: *mudrA,* the putative transposase, and *mudrB,* a helper gene; these are translated into two proteins: MURA and MURB, respectively [15]. *Mu*-like elements (MULEs) containing *mudrA* homologs and long terminal inverted repeats are widespread among both monocot and dicot angiosperms [16–20]. Any given species can have multiple, distinct subfamilies of MULEs [17].

Previous analysis of DNA gel blots and the sequences of a portion of MULEs from a number of grass species revealed a distinct lack of congruence between species phylogenies and that of their MULEs [17], consistent with horizontal transfer. However, given the presence of multiple MULE subfamilies in most grass genomes, a lack of congruence between host and MULE phylogenies would be expected if particular MULE subfamilies were lost from some plant lineages but not from others. Thus, although suggestive, incongruence between transposon and host species phylogenies cannot be expected to conclusively demonstrate horizontal transfer. Our criteria for unequivocal evidence of horizontal transfer of MULEs require that sequences from two species be too similar in noncoding regions to be reasonably expected to have diverged at the same time as their respective host species. Based on such evidence, we report horizontal transfer of a nuclear-encoded transposable MULE between two Gramineae (grass) lineages: the Panicoid and the Bambosoid subfamilies. Extensive phylogenetic and paleontologic evidence suggests that these subfamilies diverged between 30 and 60 million years ago [21–23]. The degree of similarity in noncoding and synonymous sequence between MULEs in these groups is inconsistent with this divergence time.

## Results

We have isolated portions of MULEs from several species of *Setaria* (millets of the tribe Panicoideae) and compared them with those in publicly available databases and with sequences we have previously obtained [17]. Some of these sequences are unexpectedly similar to a small group of rice MULEs. The observed degree of similarity in noncoding and synonymous sites is much higher than one would predict given the time separating *Setaria* and rice.

A total of 27 sequences from eight species of the genus *Setaria* were obtained. All 27 sequences encoded potential products with similarity to the MURA protein from maize (data not shown). Nineteen nonredundant sequences were subjected to more detailed analysis. Phylogenetic analysis revealed that all of the sequences fell into previously defined class II or class III groups of the *MuDR* family of MULEs (Figure 1). These classes of elements are phylogenetically distinct from the other known functional MULEs such as *Jittery* in maize [17] and *AtMu1* in Arabidopsis [24]. As has been previously observed with other species, a single species of *Setaria* may have elements from more than one class of MULEs, consistent with previous observations that multiple, paralogous families of MULEs can exist within a single genome [15].

**Figure 1:**
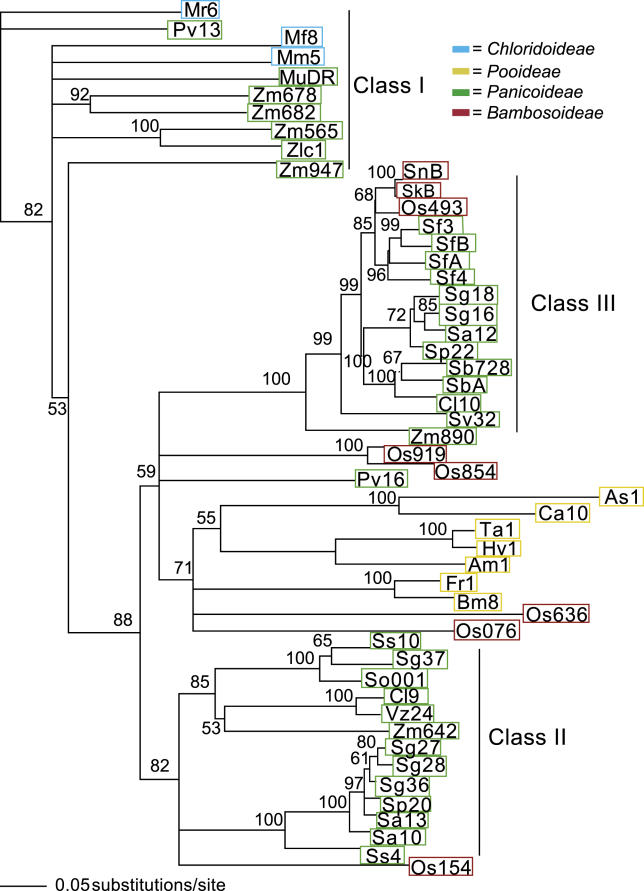
Phylogenetic Analysis of Various *mudrA*-Homologous Sequences from Grasses Representative of Several Major Subfamilies of Grasses Species designations are as follows: Zm, *Zea mays* (MuDR is *mudrA*); Zl, *Zea luxurians;* Sv, *Setaria viridis;* Sa, *S. anceps;* Sf, *S. faberi;* Ss, *S. sphacelata;* Sg, *S. glauca;* Sp, *Setaria palmifolia;* Sb, *Sorghum bicolor;* Cl, *Coix lacryma;* Os, *O. sativa;* Pv, *P. virigatum;* Vz, *Vetevaria zizanoides;* Mr, *Muhlenberia rigens;* Mm, *Muhlenbergia macroura;* So, *Saccharum officinarum;* Sk, *Shibataea kumasaca;* Sn, *Sinarundinaria nitida;* As, *Avena sativa;* Ca, *Calamagrostis acutifolia;* Ta, *Triticum aestivum,* Hv, *Hordeum vulgare;* Am, *Ammophila arenaria;* Fr, *Festuca rubra;* Bm, *Briza maxima.* Numbers represent individual clones from each species, or the last three digits of the accession number if the sequence was obtained from NCBI. Colored blocks indicate major subfamilies: green, *Panicoideae*; blue, *Chloridoideae*; yellow, *Pooideae*; red, *Bambusoideae*. Branch lengths are proportional to distance, as indicated by the scale bar. Bootstrap support is as indicated for each branch.

MULE sequences from *Setaria italica* (foxtail millet, not shown) and the closely related *Setaria faberi* (giant foxtail, “sf” in Figure 1) are strikingly similar (approximately 90% identical over 585 bp) to an element present on Chromosome 5 of the nuclear genome of *Oryza sativa* ssp. *japonica*. This element is designated *Os493.* A nearly identical element (98.9% nucleotide identity) is also present in *O. sativa* ssp. *indica*. Based on the observation that the sequences flanking the transposon insertion in both of these *O. sativa* ssp. are the same, we conclude that this element is present at the same position in each of these subspecies. *Os493* has similarity to MURA from maize from amino acid 37 to amino acid 730 of the 823–amino acid maize protein. *Os493* is flanked by the 9-bp direct repeats typical of *Mu* transposon insertions. A second, truncated element, *Os072,* is located on Chromosome 1 in *japonica* and is also present at the same position in the *indica* genome. Counting indels as single events, this element is 96% identical to *Os493.* Like the full-length elements, this element is flanked by the same 9-bp flanking duplications in both subspecies. A final truncated element on Chromosome 5, *Os408,* is present in the *japonica* genome but is absent in sequences available from the *indica* genome. This element is 94% identical to *Os493.* These are the only rice elements with significant (>80% nucleotide identity over 585 bp) similarity to the *Setaria* elements. blastn (Nucleotide-Nucleotide Basic Local Alignment Search Tool [BLAST]; National Center for Biotechnology Information [NCBI], Bethesda, Maryland, United States) searches using the full-length element from rice reveal that all other significant blast hits (e value <10^−4^) to *Os493* that are not members of the genus *Oryza* are to sequences from maize, sorghum, or sugarcane, all of which, like *Setaria,* are Panicoid grasses of the tribe Andropogoneae. To confirm the presence of this class of elements in our rice sample, we used primers specific to *Os493* to amplify several of them. One product was identical over 794 bp to *Os072,* a close relative of *Os493*. A second product was 98% identical to *Os493* (data not shown).

To understand more fully the overall distribution of this class of MULEs within the grasses, Southern blot hybridization was employed using a portion of the *mudrA* homolog from one *S. faberi* element (designated *Sf3*) as a probe to a wide variety of species representing the major subfamilies of the grasses (Figure 2). Hybridization and washing of the probe were performed at high stringency (see Materials and Methods); we have empirically determined that this degree of stringency will not detect sequences less than 87% identical to the probe (see below). The results revealed a strikingly discontinuous distribution of this class of elements. As expected based on our sequencing and database searches, it is present in a subset of the Panicoideae, with the strongest hybridization occurring with DNA from *Setaria* sp., although some species, such as *Setaria sphacelata,* hybridized poorly to the probe. Note that the probe did not detect any MULEs in *Setaria anceps,* which has an element that is 87% identical to the probe, thus defining the limit of detection for this probe. At this level of stringency, we did not detect signal from any species of the subfamilies Chloridoideae or Pooideae and only weak signal from Panicoid grasses such as sorghum and maize. As expected, DNA from rice hybridized well to the probe, and the size of the BamHI fragment is that expected for *Os072* (the predicted size of the *Os493* fragment would be too large to transfer onto this blot). Two other Oryzoids, *Ehrharta erecta* and *Zizania latifolia,* did not hybridize. Interestingly, three species of bamboo indigenous to East Asia (*Shibateae kumasaca, Fargesia nitida,* and *Phyllostachys vivax*) hybridized well to the probe, but three species of bamboo indigenous to Central America (*Chusquea montana, Chusquea quila,* and *Otatea acuminata*) did not. Primers designed from the *Setaria* element were used to amplify sequences from *Shibateae kumasaca* and *Fargesia nitida;* sequencing of these products confirmed that they are highly similar to both the rice and the *Setaria* sequences. Indeed, each was 93% identical over 641 bp to the *Setaria Sf3* element and 95% identical to rice *Os493.* Despite the higher similarity of these sequences to those in *Setaria,* we focused our analysis on a comparison between rice and *Setaria* because of the availability of the fully sequenced rice genome.

**Figure 2:**
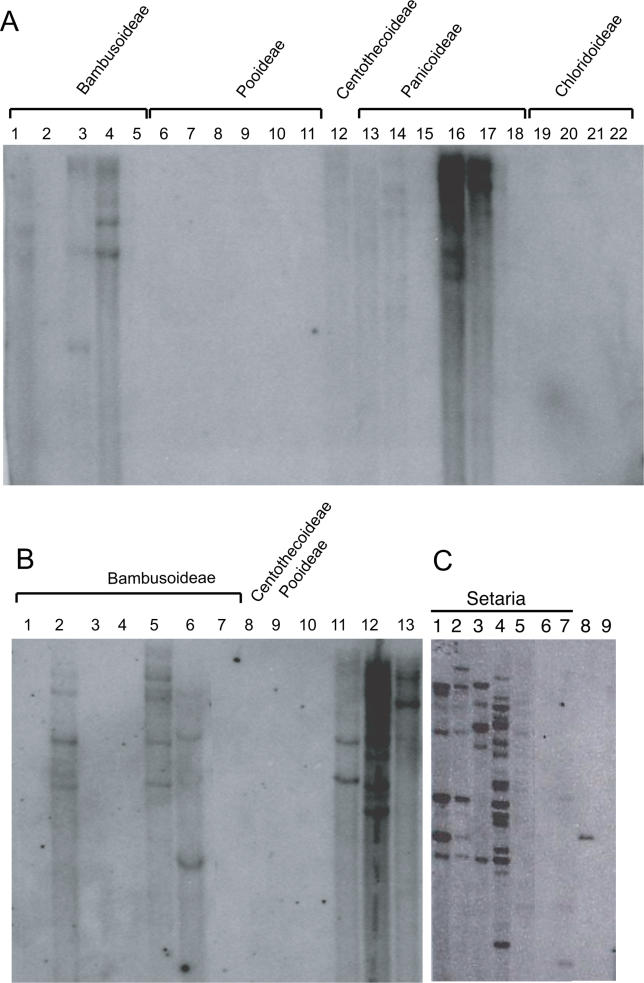
DNA Gel Blots of Representative Species from All Major Subfamilies of the Grasses All blots were probed with a fragment of MULE *sf3* from *S. faberi.* Subfamilies (i.e., Pooideae) are as indicated. (A) 1, *Shibataea kumasaca;* 2, *Ehrharta erecta;* 3, *Oryza sativa indica;* 4, *Oryza sativa japonica;* 5, *Zizania latifolia;* 6, *Nardus stricta;* 7, *Diarrhena japonica;* 8, *Brachypodium sylvaticum;* 9, *Phalaris aquatica;* 10, *Hordeum vulgare;* 11, *Triticum aestivum;* 12, *Chasmanthium latifolium;* 13, *Andropogon gerardii;* 14, *Sorghum bicolor;* 15, *Tripsacum dactyloides;* 16, *S. faberi;* 17, *S. italica;* 18, *Zea mays;* 19, *Muhlenbergia rigens;* 20, *Eleusine indica;* 21, *Boutelova curtipendula;* 22, *Cortaderia jubata.* (B) 1. *Zizania latifolia;* 2, *Shibataea kumasaca;* 3, *Chusquea montana;* 4, *Chusquea quila;* 5, *Sinarundinaria nitida;* 6, *Phyllostachys vivax;* 7, *Otatea acuminata;* 8, *Zeigotes* sp.; 9, *Brachypodium sylvaticum;* 10, *Zizania latifolium;* 11, *O. sativa (japonica);* 12, *S. faberi;* 13, *S. italica.* (C) 1, *S. italica;* 2, *S. italica;* 3, *S. viridis;* 4, *S. faberi;* 5, *S. glauca;* 6, *S. anceps;* 7, *S. sphacelata;* 8, *O. sativa;* 9, *Zea mays.*

To extend our analysis to include both coding and noncoding sequences, we obtained most of the complete sequence of a single MULE from *S. faberi.* Using PCR primers designed from the rice MULE terminal inverted repeats, it was possible to amplify and sequence a complete copy of a *Setaria* element with the exception of the 20 bp on both ends that were used as primers. This nearly complete element is designated *Sf4. Sf4* differs from the *Setaria* elements obtained in the first round of amplifications, but it retains significant similarity to *Os493* (see Figure 1). The presence of frameshift and/or stop codons in both the *Setaria* and rice MULEs (Figure 3) suggests that neither element is currently functional. However, both sequences contain long terminal inverted repeats (approximately 176 bp) and clear nucleotide similarity to the *mudrA* gene of *MuDR.* A region near the 5′ terminal inverted repeats (nucleotides 222–560 in *Os493*) has a number of deletions or insertions in one of the two elements, including a 41-bp deletion in the *Os493* element. A second region near the 3′ terminal inverted repeats (nucleotides 3350–3568 in *Os493*) has reduced homology (only 58% identity). Not counting these two regions and counting indels as single events, overall nucleotide identity of the two MULEs is 88% over 3.8 kb. This is a degree of similarity comparable to that of a well-conserved host gene. For example the *waxy* gene from *S. italica* shares 88% identity over 1.6 kb of open reading frame with *waxy* from rice (Table 1).

**Figure 3:**
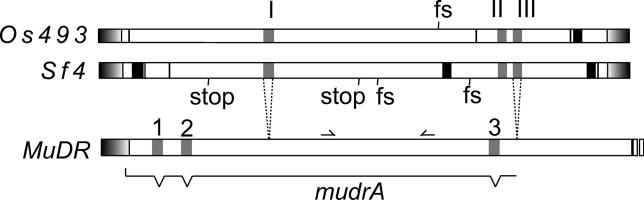
Schematic of the MULE Elements from Rice (*Os493*) and *Setaria faberi* (*Sf4*) Black blocks represent regions deleted in one or the other sequence. Grey blocks represent putative introns, which are numbered in the *Setaria* and rice elements with Roman numerals I–III. The second intron is collinear with the third intron in *mudrA* from maize. The *mudrA* introns are numbered 1–3. Shaded blocks on the ends of the elements indicate the terminal inverted repeats. For comparison, the 5' end of *MuDR* from maize (which includes the *mudrA* gene) is also included. Note that only the third intron of the *mudrA* gene and the third intron in the rice and *Setaria* is present in all three elements. The position of the RF2 and RR2 PCR primers are as indicated on the *MuDR* element. Stops and frameshifts in the *Setaria* and rice element (assuming introns are spliced) are at the positions indicated. Dotted lines connecting *MuDR* to *Sf4* indicate regions of similarity.

**Table 1:**
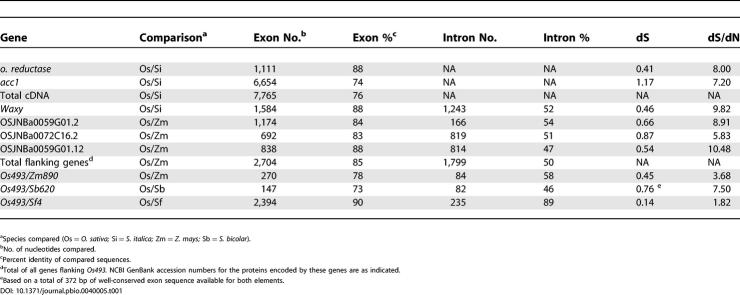
Comparisons of Coding and Noncoding Sequence Similarities

Due to the long period of time separating *Setaria* and rice (roughly 50 million years), high sequence similarity over more than 3 kb can only be due to selection or horizontal transfer; the species are distantly related such that long sequences not under selection are no longer recognizably homologous [25]. Further, conservation of noncoding sequences is far lower in plants than it is in animals, despite very similar neutral mutation rates [26].

In order to directly compare genes from *Setaria* and rice, we have examined several nuclear-encoded genes from these two species. Only a few such genes have been sequenced from the genus *Setaria,* mostly from *S. italica.* We have obtained the complete sequence of two nuclear-encoded cDNAs from *S. italica* that have clear homologs in rice: acetyl-coenzyme carboxylase *(acc1)* and oxidoreductase *(o. reductase)* (Table 1). The total cDNA coding sequence available was 7,765 bp (Table 1). The total untranslated upstream and downstream sequence (sequences immediately adjacent to the start and stop codons) from these genes was 1,203 bp (data not shown). When comparing rice with *S. italica,* these genes show an overall average identity of 76% in coding sequences (Table 1) and only 40% in the untranslated sequences after alignment (data not shown). The corrected frequency of nonsynonymous substitutions for these genes ranges from 0.41 to 1.17, and the corrected frequency of synonymous substitutions ranges from 0.05 to 0.16. Importantly, the ratio of synonymous to nonsynonymous substitutions per site (dS/dN) for these genes was 7.2 and 8.0, consistent with selection for function at the amino acid level (Table 1). The dissimilarity of the untranslated sequences immediately upstream and downstream of the coding sequences as well as the divergence of synonymous sites is consistent with the absence of selection on these sequences during the roughly 50 million years since the divergence of the *Setaria* and rice lineages. Very little genomic sequence is available for any *Setaria.* However, the complete genomic copy of *S. italica waxy* gene is available. The exons of this gene were 88% identical to the rice *waxy* gene over 1,584 bp. In contrast, the introns shared only 52% identity over 1,243 bp (Table 1), which is comparable to the frequency of substitutions in UTR and synonymous sites in this gene and the cDNA sequences (data not shown).

Unlike other genes in rice and *Setaria*, the high degree of homology of the MULEs is not the result of selection at the level of amino acid sequence. This can be inferred from analysis of both introns and synonymous sites.

Because the horizontal transfer hypothesis rests heavily on the analysis of the MULE introns, we will describe them in some detail. The MULEs in rice and *Setaria* contain three predicted short introns at the same locations in each element (Figure 4; see also Figure 3). The first predicted intron represents an insertion relative to *mudrA* in maize, which lacks this sequence. The intron is 82 bp long in the rice element and 78 bp long in the *Setaria* element. Successful splicing of this putative intron would maintain the reading frame of the rice element and precisely remove the insertion from both MULEs (Figure 4A). Left unspliced, the intron in both MULEs would result in the introduction of two stop codons and would alter the reading frame of the putative transcript of the rice element. Although no homologous cDNAs are available, an exon trap database, in which genomic sequences are expressed to examine splicing patterns, reveals that this intron can be precisely spliced from *Os493.*

**Figure 4:**
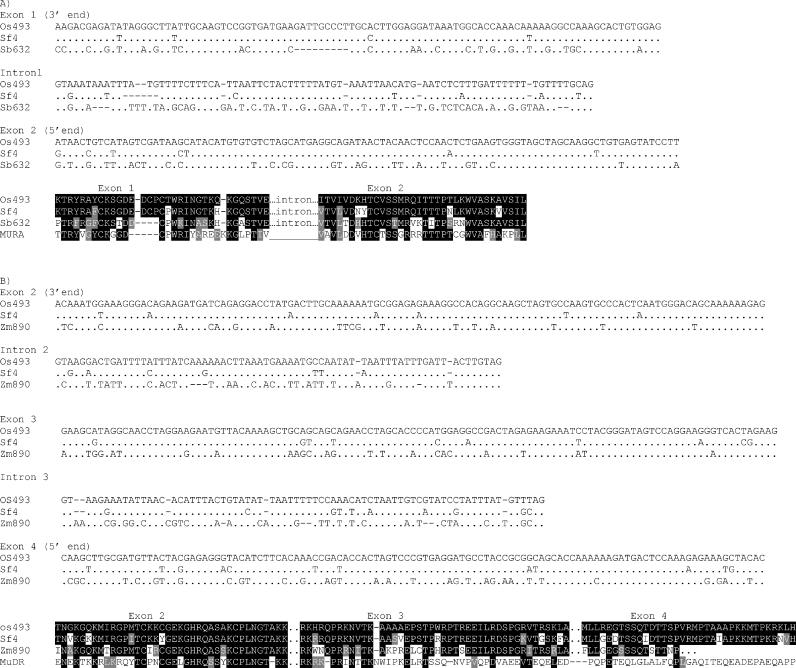
A Comparison of Coding and Noncoding Sequences from Various MULEs (A) An alignment of the nucleotide sequence of a portion of the first exon, the first intron, and a portion of the second exon from the rice, *Setaria,* and sorghum MULEs. Identical nucleotides are displayed as periods; differences are as indicated. (B) An alignment of the 3' end of exon 2, intron 2, exon 3, intron 3, and the 5' end of exon 4 from *mudrA* homologs from rice *(Os493), S. faberi (Sf4),* and a related element from maize *(Zm890).* In both panels, amino acid translations, shaded for similarity to *Os493,* are portrayed below the nucleotide alignments. MURA sequence is provided for comparison.

The second intron is in the same position as is the third intron of *mudrA* (see Figure 2). This intron is 71 bp in both the *Setaria* and rice elements. Unspliced, this intron would introduce a frameshift and several stop codons to both the rice and the *Setaria* elements. Successful splicing of this intron in the *Setaria* and rice MULEs would result in expression of a short additional region of conservation between the proteins encoded by these elements and MURA (Figure 4B). This region includes a previously identified nuclear localization signal [27]. The blastn searches identify a maize EST (AW067488) with high similarity to *Os493* and *Sf4* (78% and 77% nucleotide identity, respectively, in this region). It is much more similar to *Os493* and *Sf4* than it is to *MuDR* and is presumably related by vertical transmission to the MULE in *Setaria.* A nearly identical (99% nucleotide identity) genomic version of this cDNA, designated *Zm890,* is also available. A comparison of the genomic to cDNA sequence reveals that this intron in this maize element is spliced precisely at sites predicted for the second intron of *Os493.* A comparison of the maize cDNA to the genomic version of the sequence also reveals the presence of a third intron in this region that is also predicted to be spliced from *Os493* and *Sf4.* This intron is 74 bp in the rice *(Os493), Setaria (Sf4),* and maize *(Zm890)* MULEs. Unspliced, this intron would introduce a frameshift and at least one stop codon in all three elements. The *mudrA* gene from *MuDR* in maize lacks this intron.

When we compare the *Setaria* and rice MULEs, we find that the degree of conservation is as high in these three putative introns (89% identity over the 227-bp total in the three introns) as it is in the rest of the transposon (Figures 4 and 5). For comparison, we can use a portion of the similar element in maize *(Zm890)* for which we have both genomic and cDNA sequences. Although the exon sequences are quite similar to the rice and *Setaria* MULEs, the intron sequences retain only limited similarity (Figures 4 and 5). Overall, in the region depicted in Figure 4B that compares the maize and *Setaria* elements, which includes 348 bp of exon sequence and 145 bp of intron sequence, exon sequences are 92% identical (319 of 348) in comparing the rice and the *Setaria* elements. Together, the last two introns are 88% identical (128 of 145). In contrast, when the maize MULE *Zm890* is compared to *Os493,* although the exon sequences are 78% identical (270 of 348), the intron sequences are only 58% identical (85 of 145), consistent with selection on exon but not intron sequences.

**Figure 5:**
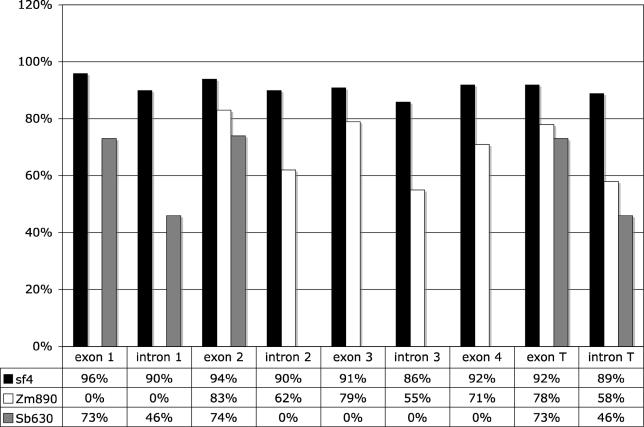
Bar Graph Displaying Percent Similarity of the Setaria *(Sf4),* Maize *(Zm890),* and Sorghum *(Sb662)* Elements with the Rice Element *(Os493)* The region analyzed includes the last portion of exon 1, intron 1, portions of exon 2, intron 2, exon 3, intron 3, and the first portion of exon 4. Although percent similarity for exon 1 and intron 1 between *Sf4* and *Os493* is shown, the corresponding region in *Zm890* is not available, nor are sequences for the last two introns of *Sb662.* The percent figure is the percent identity of each element in the specified region to *Os493* in that region. Portions in which no sequence was available are given a 0% value. T Exon and T Intron refer to the sum of exons and introns, respectively, that were compared.

A similar situation can be observed when comparing the first intron to a portion of a sorghum element *(Sb632)* available in the database (CW076632) that also has an inserted sequence at this position (see Figure 4A). While flanking exon sequences from this element are 73% identical to *Os493* (147 of 201 nucleotides), this intron is only 46% identical to that of *Os493* (38 of 82 nucleotides). In contrast, this intron is 90% identical (74 of 82 nucleotides) when comparing *Os493* with *Sf4.*

Thus, in each case where homologous sequence is available, selection appears to favor sequence similarity in exons but not in introns, except when *Os493* and *Sf4* are compared. The difference between the expected similarity of the introns in these transposons based on the *waxy* gene data (52% of 227 = 118) and the observed similarity (89% of 227 = 202) is highly significant (*p* < 0.001 using a χ^2^ test).

A common measure of selection is the ratio dS/dN [28]. When *waxy* sequences from *Setaria* and rice are compared, that proportion is 9.82. This is a typical result for a reasonably well conserved host gene from these two species since selection operates much more efficiently on nonsynonymous base substitutions. In contrast, the putative coding regions from rice and *Setaria* MULEs have a dS/dN ratio of only 1.82, consistent with very weak selection (Table 1). This is further supported by the observation that the dS/dN ratio for the comparison between the *Os493* and *Zm890,* two sequences that we hypothesize diverged at the same time as their hosts, is 3.96, consistent with selection on the nonsynonymous sites in this element. Similarly, when a well-conserved portion of exon *Sb632* is compared to the same region of *Os493* (nucleotides 1546–1917), there is clear evidence of selection at the amino acid level, with a dS/dN ratio of 7.5. For comparison, when *Os493* and *Sf4* are compared in the same region, the dS/dN ratio is only 2.1 (data not shown). These data strongly suggest that although MULEs can be subject to selection at the amino acid level (for example, when comparing *Sb632* and *Os493*), the high degree of similarity between the elements in *Setaria* and rice is not due to selection at this level.

Codon bias is a possible source of selective constraint on synonymous nucleotides. A common measure of codon bias, the effective number of codons (Nc), can vary from 21 (only one codon used per amino acid) to 61 (equal use of all possible codons) [29]. The *Setaria* and rice sequences each have an Nc value of 48, consistent with only relatively moderate codon bias. We also observe that other pairs of *mudrA* homologs compared to date radically diverge at synonymous sites [17], suggesting that selection on MULEs can operate to maintain amino acid similarity but does not prevent silent substitutions. Together, these data suggest that codon bias is unlikely to explain the high degree of similarity between the *Setaria* and rice MULEs.

One possible explanation for the low sequence divergence of the rice and *Setaria* elements is that they are found in regions with a reduced mutation frequency. In order to test this hypothesis, genes flanking the *Os493* insertion in rice were compared with orthologous sequences in maize. Maize was used as a proxy for *Setaria* because it is equally distant from rice as is *Setaria,* and, in contrast to *Setaria*, there are a very large number of genomic and cDNA sequences available in maize, making the identification of true orthologs relatively straightforward. If this region were subject to a reduced mutation frequency, we would expect to observe a low degree of variation when comparing intron or silent site sequences from the genes in rice with orthologs in maize.

The closest genes in rice with recognizable orthologs in other grasses were 144 kb 5′ of the *Os493* element and 67 and 79 kb 3′ of this element. Although there were some closer putative ORFs, the lack of close homology to any known cDNA or genomic sequence made them poor candidates for comparison. Although each of these genes has at least one paralog in rice, the sequences we identified in maize were more similar to these genes than they were to their paralogs, indicating that the maize sequences are the true orthologs. The intron/exon boundaries of these genes were well supported by cDNA data as well as by protein homology.

Comparisons between the rice genes in the region surrounding *Os493* and their orthologs in maize revealed a similar frequency of base substitutions in introns and synonymous sites as was observed in our other comparisons (see Table 1). Overall, exon sequences were 85% identical (2,297 of 2,704); introns were only 50% identical (891 of 1,799) (Table 1). dS, a measure of the synonymous substitution frequency, was similar to that observed in other comparisons, ranging from 0.54 to 0.87. These data demonstrate that the region into which the *Os493* element is inserted does not exhibit a markedly reduced frequency of mutations.

In summary, the data are consistent with horizontal transfer of a MULE between two species of grass at some point well after the divergence of the Bambosoid and the Panicoid subfamilies. The degree of similarity of these two elements in noncoding sequences makes it highly unlikely that these two sequences diverged at the same time as their hosts.

## Discussion

Our conclusion that a MULE was horizontally transferred between the *Setaria* and rice lineages leans heavily on evidence that these lineages diverged roughly 50 million years ago. Fortunately, the phylogeny of the grasses has been extensively documented, using a variety of criteria, including morphology, chloroplast restriction sites, both nuclear and mitochondrial genes, and overall gene and chromosome order [23]. Paleontologic evidence suggests that the overall age of the grasses is between 55 and 70 million years [30]. The minimum divergence time for the subfamily Bambosoideae and the rest of the grass subfamilies has been estimated to be between 35 and 60 million years [21,22,31], and the genus *Setaria* has been placed with high confidence in the subfamily Panicoideae [32]. Our analysis of three pairs of homologous genes from *Setaria* and *rice* is consistent with an early divergence for *Setaria* and rice; sequences immediately upstream and downstream of the coding sequences are radically diverged, as are intron sequences in the *waxy* gene (see Table 1). Further, corrected frequencies of synonymous substitution (dS) range from 0.41 to 1.17. The nuclear gene encoding plastid acetyl-CoA carboxylase *(acc1)* is particularly revealing because it has been used extensively in phylogenetic analysis of various grass subgroups [33] (and references therein). Not surprisingly, *Setaria acc1* is much more similar to a portion of another Panicoid grass *(Panicum virgatum)* than it is to rice *acc1* (95% versus 79% identity, respectively, over 696 bp).

Although the *Setaria* and rice MULEs are 89% identical, they have a dS/dN value of only 1.82, and their introns are as similar as the rest of the transposon (see Table 1; Figure 5). Thus, in contrast to host genes, the degree of similarity between synonymous sites and between introns when comparing the *Setaria* and rice MULEs is indeed an anomaly when comparing genes from these two species.

Although it is a formal possibility that the introns identified in the rice and *Setaria* MULEs are differentially spliced, resulting in conservation of intron sequences, we find this unlikely. In order to maintain the correct open reading frame and to avoid the appearance of multiple stop codons, each of the three introns in the *Setaria* and rice MULEs must be spliced. Further, we observe that homologs of *Sf4* and *Os493*, *Zm890* and *Sb632,* maintain similarity in the exons but lose similarity in the introns, suggesting that these introns are not normally subject to selection in this class of MULEs. Together, these data strongly suggest that all three putative introns are historically functional, suggesting that conservation within these introns when comparing the *Setaria* and rice MULEs is probably not due to differential splicing.

Our analysis of genes near the MULE *Os493* insertion in rice demonstrates that this region does not exhibit a particularly low frequency of base substitutions; the introns in these genes exhibit little homology to those of their maize orthologs. Of course, it is still a formal possibility that the region *immediately* adjacent to the MULE exhibits a particularly low mutation frequency. However, the absence of sequences similar to the regions immediately adjacent to *Os493* in any database suggests that these sequences are not well conserved. Further, it is worth noting that both the *Setaria* and the rice elements are located at a number of different chromosomal positions. Since all of these elements are quite similar to each other in noncoding and silent sites, they would all have to be located in regions with reduced mutation frequencies, which does not seem likely.

Overall, our data clearly indicate that the degree of similarity we observe between the *Setaria* and rice MULEs is not due to selection, nor does it appear that either MULEs in general or the regions in which these particular MULEs are found have a particularly low mutation rate; the only reasonable alternative explanation is horizontal transfer.

The mechanism of transfer between ancestors of the Bambusoid and the Panicoid subfamilies is a matter of pure speculation. Based on the divergence of the MULE sequences available and the presence of this class of elements in both rice and some bamboos, the transfer probably occurred several million years ago, although there may well be a *Setaria* MULE that we have not yet identified that is even more similar to the rice elements than the ones we have identified. The presence of the same MULE at the same chromosomal position in both the *indica* and *japonica* varieties demonstrates that the transfer happened before their divergence, which has been dated at roughly 1 million years ago [34]. The presence of very similar MULEs in a number of bamboo species suggests that the transfer may have occurred prior to the divergence of rice and bamboo. However, the rice and bamboo sequences are remarkably similar (95% identity between species that diverged at least 36 million years ago [31]). Further, the pattern of hybridization appears to have more to do with physical, rather than phylogenic, proximity [35]. Two Old World members of the subtribe Bambusinae (*Shibataea kumasaca* and *Phyllostachys vivax*) hybridize well to the probe, but so does the Old World bamboo *Sinaruninaria nitada,* a member of the subtribe Arundinariinae. Indeed, sequence analysis reveals that the MULEs from *Shibateae kumasaca* and *Sinaruninaria nitada,* members of different subtribes, are 99% identical (715 of 722). However, none of the three New World bamboos hybridize to the probe, including the two *Chusques* and *Otatea acuminata,* all of which are members of the subtribe Arundinariinae. Together, these data suggest that there likely were additional horizontal transfer events between these bamboo lineages as well.

The ancestors of *O. sativa* [36] and *S. italica* and *S. faberi* [37] all arose in southeast Asia, suggesting (although by no means proving) physical proximity at the time of transfer, and *S. faberi* is now a common weed next to rice fields in central China (personal observation). Both rice [38] and *Setaria* [39] are obligate self-fertilizers, but both can outcross at a low frequency. There is no evidence, however, that these rather distant subfamilies can intercross.

The possibility of horizontal transfer of genetic material between plants has been a subject of considerable interest to those concerned about the escape of transgenes from an intended species to another species. Often, the concern is related to movement of genes from plants to associated microbial species [40], which has in fact been observed to occur under laboratory conditions [41], but movement between plant species is also a valid concern given recent evidence for frequent transfer of mitochondrial genes between plant species [6,8]. Certainly, there is ample evidence for gene transfer between cultivated crops and their wild relatives via hybridization [42], and even very wide crosses (albeit with heroic efforts) are possible between quite distantly related plants [42]. Our data suggest that in addition to mitochondrial encoded genes, nuclear-encoded plant transposons have been transferred as well. Given the phylogenetic distances involved, we suggest this particular transfer was mediated via a vector of some kind.

It is worth noting that there are several features of transposon behavior that make them particularly prone to horizontal transfer. Transposable elements have the capacity to insert themselves into the chromosomes of possible vectors and, subsequently, into host chromosomes. Subsequent to transfer, they can spread rapidly throughout a given species, as is evidenced by the rapid spread of *P* elements in *Drosophila melanogaster* [43]. Thus, it is not surprising that many examples of horizontal transfer of transposons have been identified. However, it is also worth noting that the elements in both *Setaria* and rice appear to be inactive, as are the vast majority of all transposable elements in most species. This is likely related to the capacity of genomes to recognize and epigenetically inactivate foreign DNA from a variety of sources [44]. The problem of dealing with invasive DNA is, then, hardly a new one, and it is one that most organisms appear to be quite competent to deal with; indeed, maintaining transgene activity in the face of endogenous mechanisms evolved to silence invading DNA is a major problem for genetic engineers [45]. Without the intrinsic invasive properties of transposable elements, it is likely that other genes, such as transgenes, could effectively invade a new species only to the extent that they provide clear selective benefits to the recipient species.

What is perhaps most surprising about these results is that they are not more common. Plants are far more likely to undergo interspecific crosses than are animals [42], and, unlike animals, plants do not sequester their germ line. A vector-mediated transformation event that occurs in the vegetative or floral meristems can potentially be transmitted to the next generation. Thus, it is remarkable that these results represent the first well-documented case of horizontal transfer of nuclear genes between plants, particularly given the observation that plant mitochondrial genes appear to be particularly prone to horizontal transfer. Given the vast quantity of sequence data now available for a variety of plant species, the time would seem ripe for a comprehensive search for horizontally transferred plant genes.

## Materials and Methods

### Biological materials

*Setaria* samples were obtained from a variety of sources. Collection sites and accession numbers are available on request. Samples from all other species are as described in Lisch et al. [17].

### Amplification and cloning

PCR amplification of the conserved portion of the *mudrA* transposase in a number of *Setaria* sp. was performed using PCR primers RF2: CTTAGTGTAAACTCAACTGC and RR2: GGCTTGCCAGTGTGTTGCCA . These primers are anchored in two well-conserved portions of the *mudrA* gene and span the region of nucleotides 1751–2369 of the complete *MuDR* element from maize [17]. Amplification products were cloned into the TA vector (Invitrogen, Carlsbad, California, United States), and multiple independent clones from each species were sequenced. In each case, both strands were sequenced. Each species gave rise to multiple products with few repeats, suggesting that only a subset of amplified products were sequenced from any given species. A total of 19 sequences from eight species of *Setaria* were subjected to detailed analysis. Clones from several species were strikingly similar to sequences in rice. One clone from *S. faberi* was found to be 90% identical over 585 bp to a portion of rice Chromosome 5. The terminal inverted repeats of that sequence were identified using blastn. The borders of the insertion were confirmed by identification of the host direct repeat sequence, a short sequence of DNA that is duplicated upon insertion. This complete element was designated *Os943.* A single primer specific to the first 20 bp of *Os493* in rice ( GAGAAAATTGCAATTATAGG) was synthesized and used to amplify a similar complete element from *S. faberi.* One product of 3.85 kb was cloned into a TA vector (Invitrogen), and both strands of the complete sequence of a single clone were obtained using a series of overlapping sequencing primers. Products from each overlapping sequencing reaction were assembled using SeqMan (DNAStar, Madison, Wisconsin, United States). Although the sequence of this nearly complete element was different from that obtained in the first round of amplification (using primers RF2 and RR2), it retained a high degree of similarity to *Os493.* All samples were subjected to 35 rounds of amplification, gel isolated, and purified using the QIAquick Gel Extraction Kit (Qiagen, Valencia, California, United States) and subcloned using the TOPO TA Cloning kit (Invitrogen). The University of California, Berkeley sequencing facility performed sequencing using an Applied Biosystems sequencer (Foster City, California, United States).

### Sequence analysis

Sequences were aligned to each other using ClustalW (http://www.ebi.ac.uk/clustalw/index.html) [46] or using blastn if the sequences were highly similar. The rice and *Setaria* sequences were also compared to MURA using tblastn (NCBI). This alignment was used to determine the correct reading frame for both the rice and *Setaria* sequences, which were then modified to maintain that reading frame for subsequent analysis of codon usage and bias. Putative introns were identified using SplicePredictor (http://bioinformatics.iastate.edu/cgi-bin/sp.cgi) and GeneSeqer (http://www.maizegdb.org/geneseqer.php) as well as comparisons to available cDNA and gene trap sequences and to MURA. These methods use a Bayesian model to predict the placement of intron sequences based on a large database of empirically determined splice sites in plants [47]. The last two putative introns (nucleotides 2809–2878 and 2996–3069 in *Os493*) gave high scores (*p* > 0.9) for donor and acceptor sites using SplicePredictor and GeneSequer and were missing in a spliced maize cDNA (AW067488). The first intron (1122–1203) also gave a high score using SplicePredictor and GeneSeqer and, when spliced, restored continuity with the maize MURA protein sequence. The fact that the inserted sequence precisely corresponds to predicted mRNA splice sites strongly suggests that this sequence is in fact an intron. Two other high-scoring putative introns (547–625 and 1978–2056) were excluded from the analysis because they lacked both cDNA and protein homology support.

A portion of recognizable open reading frame (2,394 bp) from MULEs *Os493* and *Sf4* was examined for differences in synonymous and nonsynonymous changes using the SNAP (Synonymous/Nonsynonymous Analysis Program) tool in the HIV Sequence Database (http://www.hiv.lanl.gov) [48] after manual correction of frameshifts or small deletions. This program uses the algorithm devised by Nei and Gojobori [28]. Codon bias as determined by the Nc value was computed using CodonW (http://bioweb.pasteur.fr/seqanal/interfaces/codonw.html) [29]. Multiple sequence alignments used to generate the phylogenetic tree in Figure 1 were performed using the ClustalW server available at European Bioinformatics Institute (http://www.ebi.ac.uk/clustalw/) with default parameters. The nucleotide sequences used corresponded to a 583-bp portion of *MuDR* (nucleotides 1171–2349). Included in the phylogenetic analysis are sequences previously obtained at this laboratory [17] as well as the best blastn hits to each of the sequences obtained in this study. In order to comprehensively search the database, each available sequence was blasted against all available databases, and the top two hits were then added to the dataset. This process was then repeated. Close duplicates were excluded. The phylogenetic tree in Figure 1 was generated based on the distance method, using PAUP* Version 4.0b8 (Sinauer Associates, Sunderland, Massachusetts, United States) using BioNJ with the Kimura 2 parameter distance correction and with 1,000 bootstraps. Branches with less than 50% bootstrap support were collapsed.

### Identification of rice homologs of *S. italica* cDNAs

Complete cDNA sequences from *S. italica* were obtained from NCBI. These sequences were used as queries to databases including all publicly available rice sequences, including the complete draft of the *indica* subspecies. The most significant blast hits were retrieved and aligned with the *Setaria* sequences using ClustalW. Coding versus noncoding sequences were determined using annotation available for each gene or by using blastx to compare with known protein sequences. To determine similarity of introns, noncoding upstream and downstream UTRs, the sequences of each gene (or portion of gene) pair were aligned using ClustalW and the percent identity calculated.

### Identification of genes near the *Os493* and their orthologs in maize

The *Os493* insertion was located using the Gramene database (http://www.gramene.org/, which allows visualization of large contiguous regions of the rice genome, along with associated significant BLAST hits to a variety of other databases, including cDNA and genomic information from rice, maize, and other grasses. The region in rice flanking *Os493* can be visualized at (http://www.gramene.org/japonica/contigview?highlight=&chr=5&vc_start=2800000&vc_end=3065000&x=44&y=11). The NCBI accession numbers for the corresponding proteins are as indicated in Table 1. Genes in rice were chosen for comparison with maize if the following criteria were met: (1) the rice gene had multiple hits to rice cDNA sequences (supporting theoretical intron/exon boundaries), (2) the rice gene matched a large number of maize and other genomic and cDNA sequences, (3) the gene was not a transposon, and (4) the hit to maize was better than it was to any rice orthologous sequence. When possible, maize genomic matches were combined using DNAStar, SeqMan program (Lasergene) in order to obtain as much contiguous maize sequence as possible. After assembly, exon and intron sequences from each rice gene were independently aligned with the orthologous maize exons and introns using either blastn (for exons, which had a high degree of similarity) or ClustalW (http://www.ebi.ac.uk/clustalw/index.html) (for introns, which could not be aligned using blastn). A summary of the results of these alignments is presented in Table 1.

### DNA gel blotting

DNA extraction and gel blotting were performed as described in Lisch et al. [17]. The DNA was digested with BamHI. Washes were performed at 65° C in 0.2× SSPE and 0.2% SDS for a total of 1 hr. The probe used was a 585-bp fragment of a *mudrA*-homologous sequence obtained from *S. faberi* (*Sf3*)*.*

## Supporting Information

### Accession Numbers

The GenBank (http://www.ncbi.nlm.nih.gov/Genbank) accession numbers for rice sequences in which the MULEs described in this paper are found are *Os493* in *O. sativa* ssp. *japonica* (AC093493, nucleotides 152975–156800) and *O. sativa* ssp. *indica* ( AAAA01000154, nucleotides 41163–45085), a spliced version of *Os493* (CL970142), *Os072* (AP001072, nucleotides 86139–89558), *Os650* ( AAAA01004650, nucleotides 3332–6752), and *Os408* (AP003408, nucleotides 40419–41521). The accession numbers for portions of other MULES described in this paper are *Zm890* (CG270890) and *Sb632* (CW076632). The accession number for the complete MULE from *S. faberi* designated *Sf4* that was sequenced by this laboratory is DQ287976.

The accession numbers for the genes and gene products discussed in this paper are MULE EST similar to *Zm890* (AW067488), *waxy* from *S. italica* (AB089141), *waxy* from *O. sativa* (AF515483), *acc1* from *O. sativa* (NM_196052), *acc1* (acetyl-coenzyme A carboxylase) from *S. italica* (AY219174), *acc1* from *O. sativa* (NM_196052), *acc1* from *P. virgatum* (AF342959), oxidoreductase from *S. italica* (AY266141), oxidoreductase from *O. sativa* (AK067136), *waxy* (granule-bound starch synthase) from *S. italica* (AB089141), and *waxy* from *O. sativa* (AF515483). The accession numbers for proteins encoded by genes flanking *Os493* in *O. sativa* are as indicated in Table 1.

## Abbreviations

BLAST: Basic Local Alignment Search Tool
bp: base pair
MULE: *Mu*-like element
NCBI: National Center for Biotechnology Information
